# In Vitro Propagation Method for Production of Phenolic-Rich Planting Material of Culinary Rhubarb ‘Malinowy’

**DOI:** 10.3390/plants10091768

**Published:** 2021-08-25

**Authors:** Agnieszka Wojtania, Monika Mieszczakowska-Frąc

**Affiliations:** 1 Department of Applied Biology, The National Institute of Horticultural Research, Konstytucji 3 Maja 1/3 Str., 96-100 Skierniewice, Poland; 2 Fruit and Vegetable Storage and Processing Department, The National Institute of Horticultural Research, Konstytucji 3 Maja 1/3 Str., 96-100 Skierniewice, Poland; monika.mieszczakowska@inhort.pl

**Keywords:** anthocyanins, *meta-*topolin, micropropagation, *Rheum*, soluble sugars, sucrose concentration

## Abstract

Culinary rhubarb is a popular vegetable crop, valued for its long, thickened stalks, very rich in different natural bioactive ingredients. Tissue cultures are a useful tool for vegetative propagation of virus-free rhubarb plants and rapid multiplication of valuable selected genotypes. The aim of this study was to develop an effective method for in vitro propagation of selected genotypes of Polish rhubarb ‘Malinowy’ characterized by high yield and straight, thick and intensive red stalks. Identification and quantification of anthocyanins and soluble sugars by the HPLC method in shoot cultures and ex vitro established plantlets were also performed. Shoot cultures were established from axillary buds isolated from dormant, eight-year-old rhizomes. Effective shoot multiplication of rhubarb ‘Malinowy’ was obtained in the presence of 6.6 µM benzylaminopurine or 12.4 µM *meta*-topolin. Both cytokinins stimulated shoot formation in a manner that depended on sucrose concentration. Increasing the sucrose concentration from 59 to 175 mM decreased the production of shoots and outgrowth of leaves by 3-fold but enhanced shoot length, single shoot mass and callus formation at the base of shoots. This coincided with increased accumulation of soluble sugars (fructose, glucose) and anthocyanins-cyanidin-3-O-rutinoside (max. 208.2 mg·100 g^−1^ DM) and cyanidin-3-O-glucoside (max. 47.7 mg·100 g^−1^ DM). The highest rooting frequency (94.9%) and further successful ex vitro establishment (100%) were observed for shoots that were earlier rooted in vitro in the presence of 4.9 µM indole-3-butyric acid. Our results indicated that anthocyanin contents in leaf petioles were influenced by developmental stage. Under in vitro conditions, it is possible to elicit those pigments by sucrose at high concentration and *meta*-topolin.

## 1. Introduction

Rhubarb (*Rheum*) is a herbaceous perennial of the *Polygonaceae* family. The genus *Rheum* includes about 60 species, and most of them are native to the northern and central regions of Asia [1]. For thousands of years, *Rheum* has been cultivated in China for medicinal purposes. The dried rhizomes and roots of medicinal species (*R. palmatum* L., *R. officinale* Baill*, R. tanguticum* Maxim.) have been used to treat constipation, inflammation and ulcers [2,3].

Culinary rhubarb is popular as a vegetable crop, valued for its long, thickened stalks [4]. They are used in the production of desserts, cakes, jam, juices, fruit teas and wine. The use of rhubarb petioles for food was discovered at the beginning of the 18th century in Great Britain, but widespread consumption of rhubarb stalks began in the early 19th century. Culinary rhubarb cultivation spread to northern Europe, North America, Australia and New Zealand. Worldwide rhubarb production and consumption peaked just before the Second World War, then it came to be restricted [5]. Recently, there has been an increased interest in rhubarb production in Europe, including in Poland. This is due to the higher interest of consumers and the food industry in functional food [6]. It is known that rhubarb stalks contain useful levels of organic acids (malic acid, citric acid, fumaric acid and ascorbic acid), dietary fiber, protein, potassium, calcium and magnesium [7]. They are also rich in polyphenolic compounds, such as stilbenes, anthocyanins and flavonols, which have a range of bioactivities relevant to human health [8,9,10]. Rhubarb juice may be used as a natural food preservative. It has been shown that the addition of rhubarb juice to strawberry jams or apple purees reduced unfavorable color changes and increased antioxidant properties [7]. Consumers and the food industry prefer red stalks that are sweeter and have a higher content of polyphenolics. It has been shown that the level and composition of phenolic compounds depend on the rhubarb cultivar [11].

The culinary rhubarb called *R. rhaponticum* L. is a hybrid between rhubarb species originally brought to Europe for medicinal purposes—*R. rhaponticum*, *R. undulatum* L. (syn. *R. rhabarbarum* L.) and *R. palmatum* L. [5,12]. At an early stage, growers in England started to develop culinary rhubarb cultivars, including the still-common ‘Victoria’ and ‘Prince Albert’. Nowadays, the number of culinary rhubarb cultivars is difficult to estimate. Propagation of culinary cultivars by seed and careful selection of superior plants resulted in the development of many local cultivars with marked improvement in yield, quality and uniformity.

For successful commercial cultivation of rhubarb genotypes, a planting material of good quality is very important. In horticultural practice, seed propagation is not recommended because the obtained plants are usually not uniform and do not repeat parental traits. Plants differ in height, habit, shape and intensity of petiole color, as well as petiole size and number [13]. On the other hand, the production of rhubarb by division of crowns is limited by the low number of donor plants available and the risk of virus transfer. Rhubarb crops were found to be infected with several viruses. The most common are: *Turnip mosaic virus* (TuMV), *Arabis mosaic virus* (ArMV), *Cucumber mosaic virus* (CMV) and *Cherry leaf roll virus* (CLRV) [14,15,16,17]. A good alternative for the production of high-quality planting material is in vitro propagation of virus indexed rhubarb plants. Moreover, tissue culture of rhubarb can be a source of bioactive compounds. In rhizomes and leaf petioles of *Rheum rhabarbarum*, the accumulation of catechin, gallic acid, p-cumaric acid, rosmarinic acid, isoquercitrin and resveratrol has been reported [18]. Several articles have been published on the vitro propagation of different culinary rhubarb selections [18,19] and cultivars, including ‘Victoria’ [20], ‘Big Red’ [16] and ‘Karpov Lipskiego’ [21]. For many genotypes, bacteria contaminations, slow culture establishment, low activity of axillary buds and hyperhydricity have been reported [16,18,20]. Mentioned difficulties caused that commercial micropropagation of some valuable rhubarb selections, including ‘Malinowy’, has not been attained.

The aim of this study was to develop an efficient in vitro propagation technology for the selection of rhubarb ‘Malinowy’. Identification and quantification of anthocyanins and soluble sugars by the HPLC method in shoot cultures and ex vitro established plantlets were performed. The development of procedures for rapid in vitro clonal propagation of value rhubarb genotypes may be of great commercial value to the rhubarb industry.

## 2. Results

### 2.1. Culture Initiation

**Figure 1:**
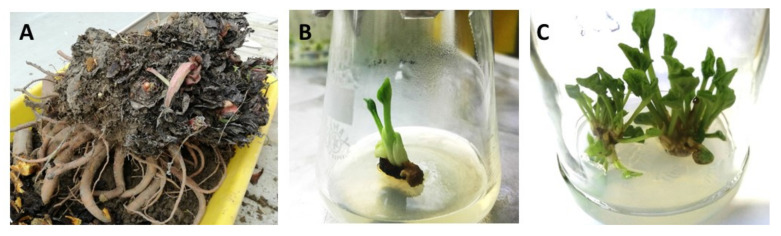
Initiation and stabilization stage of culinary rhubarb ‘Malinowy’ micropropagation: (**A**) 8-year-old mother plant collected from the plantation at the beginning of November; (**B**) initial shoot developed from axillary bud after 3 weeks of culturing in initiation medium; (**C**) the multiplied shoots at the stabilization stage.

Shoot cultures were initiated from axillary buds isolated from dormant, eight-year-old rhizomes. We obtained 51.9–67.6% uncontaminated explants that developed shoots. Contamination of initial explants of *Rheum* ‘Malinowy’ partly depended on the sanitary status of the mother plant (Table 1, Figure 1A). In MS medium without growth regulators, no explant developed shoots (Table 2). Shoot tips and axillary buds cultured in MS media supplemented with BAP and GA_3_ or NAA started to develop into single shoots in 2–3 weeks (Figure 1B). After 3–4 subcultures in the same fresh medium, new shoot formation was observed (Figure 1C). The best growth and development of initial shoots from dormant buds was observed in the modified MS medium containing 75% nitrogen salts, 4.4 µM BAP and 0.3 µM GA_3_ (Table 2). After three subcultures in media containing BAP + NAA, the developed shoots of ‘Malinowy’ were characterized by pale green leaves and a tendency to hyperhydricity, some of which died. The negative effect of auxin was enhanced when full-strength MS medium was used. It was observed that lowering the strength of nitrogen salts by one quarter in MS medium containing BAP + GA_3_ slightly enhanced new shoot formation from initial explants (Table 2).

### 2.2. Shoot Multiplication

**Figure 2:**
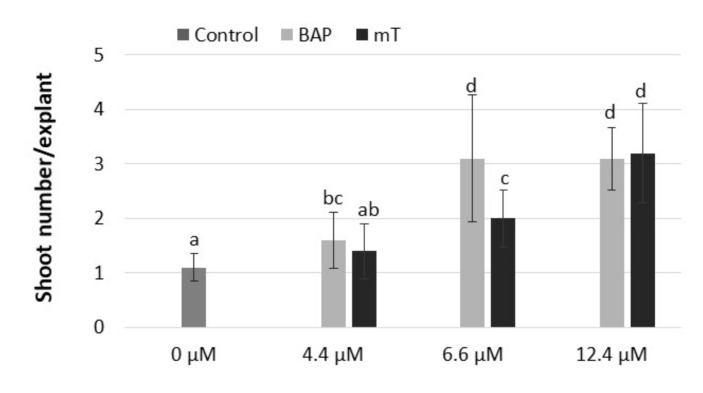
Effect of different cytokinin types (BAP, mT) and concentrations (4.4, 6.6, 12.4 µM) on shoot formation of rhubarb ‘Malinowy’ after a 4-week subculture period. Sucrose concentration in the medium was 88 mM. Medium without cytokinin was the Control. Means marked with the same letter do not differ significantly (*p* = 0.05) according to Duncan’s test.

**Figure 3:**
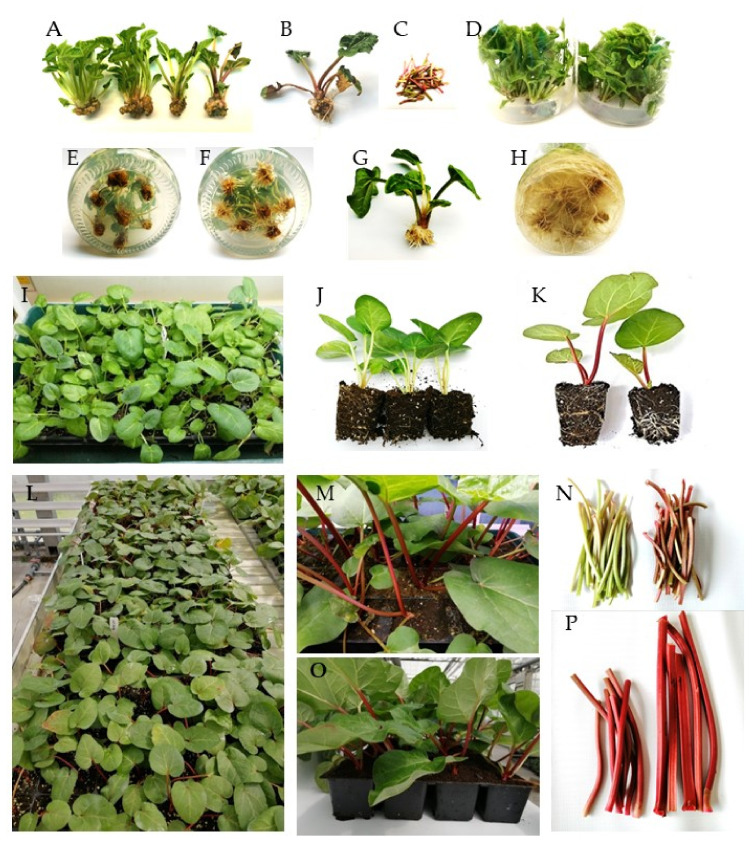
In vitro shoot formation, rooting and acclimatization of culinary rhubarb ‘Malinowy’. (**A**) Shoots multiplicated in mT medium containing different levels of sucrose (from the left: 59, 73, 88 and 175 mM); (**B**) shoots from BAP medium containing 175 mM sucrose; (**C**) leaf petioles from mT medium containing 175 mM sucrose; (**D**) shoot cultures; (**E**) roots three weeks after transfer to medium with 0.49 µM IBA; (**F**) roots three weeks after transfer to medium with 4.9 µM IBA; (**G**) shoots rooted in the presence of 4.9 µM IBA; (**H**) roots after 4 weeks in medium with 4.9 µM IBA; (**I**–**J**) plantlets four weeks after transfer to ex vitro conditions (growth room); (**K**–**L**) plantlets six weeks after transfer to the greenhouse; (**M**–**O**) plantlets nine weeks after transfer to the greenhouse; (**N**–**P**) leaf petioles after different periods of growing ex vitro.

The results of our study showed that adenine-type cytokinin (BAP and mT) was effective in the stimulation of shoot formation of rhubarb ‘Malinowy’ in vitro. It was observed that BAP was the most effective in the stimulation of shoot formation when applied at a concentration of 6.6 µM and *meta*-topolin at 12 µM (Figure 2). The next experiment showed the cytokinin effect on rhubarb shoot formation and quality significantly depended on sucrose supply. The highest multiplication rate (4.8–4.9 shoots/explant) was observed in medium with the lowest sucrose content (59 mM) and in the presence of 6.6 µM BAP or 12.4 µM mT, respectively. Increasing the sucrose supply from 59 to 175 mM in the cytokinin medium decreased the production of shoots and outgrowth of leaves by 3-fold but enhanced shoot length, single shoot mass and callus formation at the base of shoots (Table 3). Progression of shoots from a juvenile to adult phase (shoots with large, dark-green leaf petioles, single root) was also observed (Figure 3A). At 117 mM sucrose in the medium, leaf petioles started to turn red, and increased sucrose supply intensified the color of rhubarb leaf petioles under in vitro conditions. Despite the highest shoot formation rate of rhubarb ‘Malinowy’ in medium with low sucrose content (59 mM), cyclic multiplication, especially in the presence of mT, resulted in the formation of pale green shoots, with small leaf blades and a tendency to hyperhydricity and deformation. On the other hand, *meta*-topolin stimulated more juvenile shoot formation at higher sucrose levels compared to BAP. This resulted in higher shoot production at 88 mM sucrose and higher shoot quality at high exogenous sucrose supply. Shoots produced in BAP medium with high sucrose content (175 mM) showed a tendency to leaf yellowing and swelling shoot bases (Figure 3B).

### 2.3. Soluble Sugar and Anthocyanin Contents in Leaf petioles

**Figure 4:**
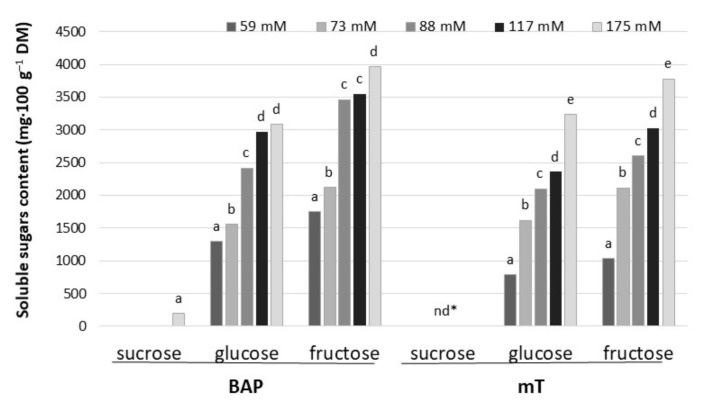
Contents of soluble sugars in culinary rhubarb ‘Malinowy’ after a 4-week subculture period in medium containing different cytokinin types: BAP (6.6 µM) and mT (12.4 µM) and sucrose levels (59, 73, 88, 117 and 175 mM); * not detectable. Duncan’s test was used independently for each type of cytokinin and sucrose concentration. Different letters indicate significant differences among sucrose treatments (*p* = 0.05).

Among tested soluble sugars, fructose and glucose were dominant in leaf petioles of rhubarb ‘Malinowy’ after a 4-week subculture period in multiplication medium. Sorbitol was not detected in rhubarb ‘Malinowy’. The content of soluble sugars in leaf petioles varied significantly depending on sucrose concentration in the medium and cytokinin type. Increasing sucrose concentration (from 59 to 175 mM) resulted in an increase in endogenous soluble sugar content by 60% and 75% in the presence of BAP and mT, respectively. Generally, BAP influenced higher fructose and glucose accumulation compared to *meta*-topolin, especially at the lowest sucrose level in the medium. In medium with BAP and high sucrose content (175 mM), the accumulation of a small amount of sucrose was also observed (Figure 4).

**Figure 5:**
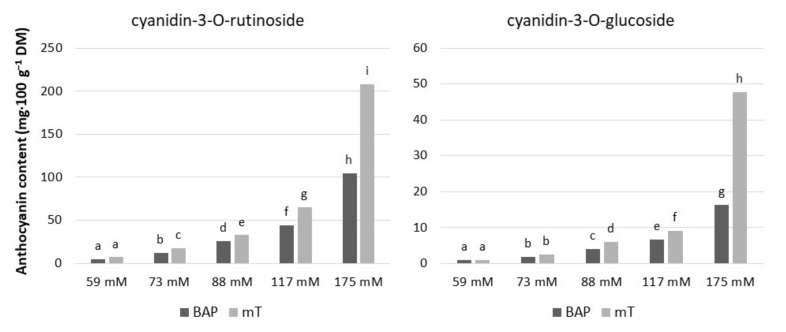
Contents of anthocyanin in culinary rhubarb ‘Malinowy’ after a 4-week subculture period in medium containing different cytokinin types: BAP (6.6 µM) and mT (12.4 µM) and sucrose levels (59, 73, 88, 117 and 175 mM). Means indicated with the same letter within cytokinin treatment do not differ significantly (*p* = 0.05) according to Duncan’s test.

We found that exogenous sucrose levels and cytokinin type had a significant effect on anthocyanin accumulation in rhubarb ‘Malinowy’ under in vitro conditions (Figure 3A and Figure 5). The most abundant anthocyanin compound was cyanidin-3-O-rutinoside (max. 208.2 mg·100 g^−1^ DM), followed by cyanidin-3-O-glucoside (max. 47.7 mg·100 g^−1^ DM). Both cyanidin levels were enhanced in rhubarb leaf petioles by increased sucrose concentration in the medium. At a high sucrose level (175 mM), the production of anthocyanins was significantly stimulated by cytokinin. It was found that *meta*-topolin resulted in 50% higher content of anthocyanins in rhubarb petioles compared to BAP (Figure 5).

### 2.4. Rooting and Acclimatization

Under in vitro conditions, roots emerged 1–2 weeks after transfer to the rooting media. As shown in Table 4, rooting of culinary rhubarb ‘Malinowy’ was significantly affected by IBA levels. In auxin-free medium, roots formed with the effectiveness of 40%. Application of IBA resulted in an increase in root formation in a concentration-dependent manner (Table 4). The best rooting response (94.9% rooting frequency; 10.7 roots/shoot) was observed in the presence of IBA at the concentration of 4.9 µM (Figure 3F). In all treatments, root length progressively increased with time (Figure 3F) and resulted in their damage during transfer to ex vitro conditions (Figure 3H).

**Figure 6:**
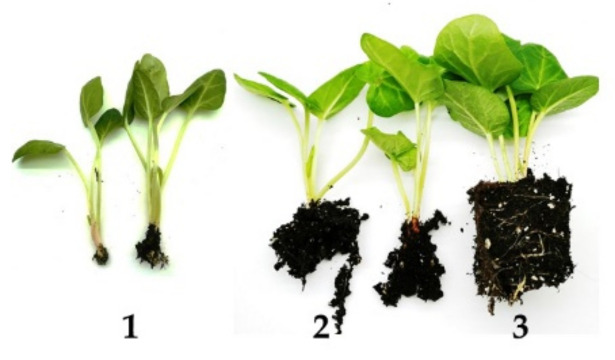
Development of the root system of culinary rhubarb ’Malinowy’ on a 1–3 scale.

We then compared the efficiency of acclimatization of rhubarb shoots rooted in vitro and directly ex vitro. The development of the root system was evaluated on a three-point scale (Figure 6). It was observed that in vitro rooted shoots rapidly developed a root system (Table 5, Figure 3J). Three weeks after transfer to ex vitro conditions, roots were visible through the walls of the peat plugs. This resulted in more vigorous growth of shoots and higher ability to tolerate low humidity compared to shoots directly rooted and acclimatized. After four weeks of acclimatization, in vitro rooted shoots were transferred to the greenhouse. Ex vitro rooted shoots needed at least 7–8 weeks to develop a root system and acclimatize. However, after this period of time, the rooting rate was poorer than after 4-week acclimatization of shoots rooted in vitro. The ex vitro establishment rate of in vitro rooted shoots was 100%, while those rooted ex vitro survived with 83%.

After transfer to the greenhouse, growth of the plantlets tended to decrease, and yellowing of the oldest leaves was observed. As shown, soluble sugar and anthocyanin contents in leaf petioles were very low at this stage of growth in the greenhouse (Table 6). On sunny days, the plantlets needed shading. After two weeks, new leaves started to develop. Six weeks after transfer to the greenhouse, the average length of leaf petioles was enhanced by 15.6% and leaf area by 47.8%. A rapid increase in leaf (petioles and blades) thickness was also observed (Figure 3K). Leaf petioles of all rhubarb plantlets turned red (Figure 3M–N). During six-week growth in the greenhouse, contents of anthocyanins and soluble sugars in the leaf petioles were enhanced by 4 and 7 times, respectively. They contained sucrose, glucose and fructose in a ratio 1:2:2. Total anthocyanin content in leaf petioles of ‘Malinowy’ was 188 mg·100 g^−1^, including 94% cyanidin-3-O-rutinoside and 6% cyanidin-3-O-glucoside (Table 6). No morphologically aberrant plantlets were found. The ten-week-old rhubarb plantlets were transferred (at the end of April) to the plantation for further observation.

## 3. Discussion

The quality and quantity of rhubarb crops are significantly dependent on planting material. Tissue cultures are a useful tool for vegetative propagation of virus-free rhubarb plants and rapid multiplication of valuable selections with the highest possible content of bioactive ingredients. For commercial micropropagation of planting material, a successful in vitro propagation protocol including all stages (initiation of aseptic culture, shoot multiplication, rooting of microshoots, ex vitro acclimatization) is very important.

Axillary bud development has proven to be the most often applied system for producing true-to-type plantlets. In the present study, dormant axillary buds of culinary rhubarb ‘Malinowy’ were used successfully for the initiation of shoot cultures. By using a two-step disinfection procedure, we obtained an average of 59.5% uncontaminated explants from eight-year-old rhizomes that developed shoots of high quality. Given the age of the rhizomes and the associated large amount of dead tissue and secreted mucus, as well as the presence of endogenous bacteria, this is a high efficiency of culture establishment. Buds of ‘Big Red’ treated with 2% sodium hypochlorite for 15 min showed 51% establishment success. Additional methods for disinfection in sodium dichloroisocyanurate (300 mg·L^−1^ for 20 min and 48 h) and 4% chlorine dioxide did not reduce the amount of contamination [16]. Clapa et al. [18], by using bleach solution of 20% (ACE-Protector) for 20 min, obtained 20% contaminated explants, but 36% were necrotic and only 44% were viable.

Data in the literature indicate that initial growth of culinary rhubarb shoots from axillary buds depends on different factors, including growth regulators, term of explant collecting, bud size and genotype [16,17,18,19,20,21]. The most important factor stimulating the activity of rhubarb buds was BAP added singly [16] or in combination with auxin [19,20]. We obtained the best growth response of dormant buds of rhubarb ‘Malinowy’ in modified MS medium containing 75% nitrogen salts, 4.4 µM BAP and 0.3 µM GA_3_. The combination of cytokinin and GA_3_ was found to increase the activity of axillary buds of many plant species, especially perennials and woody plants [22,23,24]. On the other hand, we found that NAA presence in the initial medium significantly decreased shoot quality. This might be related to the inappropriate type and concentration of auxin or lack of need of auxin for rhubarb ‘Malinowy’ shoot induction. We also observed that the negative effect of auxin was enhanced when full-strength MS medium was used. Murashige and Skoog medium with KNO_3_ (at 1900 mg·L^−1^) and NH_4_NO_3_ (at 1650 mg·L^−1^) is the most common medium for micropropagation of many horticultural plants, but these high concentrations of nitrogen salts are supraoptimal for some plant species and can induce different physiological disorders, including hyperhydricity [25]. Ogura-Tsujita and Okuba [26] reported that rhizome explants of *Cymbidium kanran* growing in low-nitrogen medium produced 55% less ethylene than those coincident with enhanced shoot production.

It has been reported that among the cytokinins tested, BAP was more effective than isopentenyladenine (2iP), kinetin and thidiazuron (TDZ) for axillary multiplication of various culinary rhubarb genotypes [19,21]. The optimal BAP concentration ranged from 2.2 to 22.2 µM according to rhubarb cultivar [16,20]. Kozak and Sałata [21] showed that ‘Karpow Lipskiego’ cultured in BAP medium was characterized by the largest number of leaves, basal tissue and fresh mass of shoots. However, the use of kinetin and 2iP enhanced the length of shoots. The genotype-dependent multiplication rate and shoot morphology of some culinary rhubarb cultivars and clones have already been observed [16]. For example, rhubarb ‘Malinowy’ cultured in BAP medium formed much lower shoots but with an enhanced number of leaves per explant compared to ‘Karpow Lipskiego’ [21]. Similar to a study on *Rheum rhabarbarum* [18], we observed that *meta*-topolin was of similar efficiency in the stimulation of axillary multiplication of rhubarb ‘Malinowy’ compared to BAP. Additionally, our study showed that both cytokinins stimulated shoot formation of rhubarb in a manner dependent on sucrose concentration. Increasing sucrose supply (from 59 to 175 mM) in the growing medium resulted in a reduction in the multiplication rate by 70% and the stimulation of mature shoot production. Sucrose inhibition of shoot formation was previously observed in different plant species, including *Helleborus niger* L., *Paeonia lactiflora* Pall*,* [24,27], *Pelargonium hortorum* L.H. Bailey [28] and *Rosa* ‘Konstancin’ [29].

It is known that sugars play important roles in in vitro cultures as energy and carbon sources and osmotic agents. They can also act as signaling molecules and/or as regulators of gene expression [30]. Sugar-mediated signals indicate carbohydrate availability and regulate metabolism with sugar usage and storage [31]. As shown in our study, the exogenous sucrose supply in cytokinin medium affected the accumulation of fructose and glucose in rhubarb leaf petioles and coincided with a reduced multiplication rate and plantlet transition from juvenile to mature phase. At high exogenous sucrose content in the medium, BAP influenced the formation of more mature shoots compared to mT. Moreover, our study showed that high exogenous sucrose content promotes the accumulation of anthocyanins in leaf petioles of rhubarb in vitro.

Anthocyanins are an important class of flavonoids that represent a large group of plant secondary metabolites. They are recognized for their diverse function in plant development and beneficial effect on human health. Generally, anthocyanin accumulation in fruits and vegetables are accompanied by their maturation and is regulated by both developmental and environmental cues [32]. Sugar-induced anthocyanin accumulation has been observed in many plant species. In *Petunia* Juss, sugars were shown to be required for the pigmentation of developing corollas [33], while in grape berry skin, sugars were found to induce most genes involved in anthocyanin synthesis [34]. Enhanced anthocyanin accumulation caused by high sucrose content in the medium has been previously reported in *Melastoma malabathricum* L. [35], *Clematis pitcheri* [36] and *Petunia* [37]. It is well known that phytohormones can also modulate anthocyanin biosynthesis by regulating the expression of genes involved in the flavonoid biosynthetic pathway [38,39]. As shown in our study, the sucrose-induced accumulation of anthocyanin in rhubarb petioles in vitro was stimulated by cytokinin, mainly *meta*-topolin. Potential of mT in stimulating the accumulation of proanthocyanidins has been previously observed for the shoot cultures of *Musa* ‘William’ [40]. The authors demonstrated that *meta*-topolin was the most effective in this process compared to other topolins and BAP.

During the last decade, *meta*-topolin has been widely used in micropropagation of many plant species, improving multiplication and rooting efficiency [41,42,43]. Our previous observation showed that the shoot of rhubarb ‘Malinowy’ treated with *meta*-topolin had higher rooting capacity than those cultured in BAP medium before rooting (data not shown). It was shown that the rooting ability of rhubarb in vitro is also dependent on genotype. For example, ‘Karpow Lipskiego’ produced 100% rooted shoots in hormone-free medium and spontaneously formed multiple roots in multiplication medium [21]. We obtained only 40% rooted shoots in auxin-free medium, but the application of IBA at 4.9 µM resulted in 100% rooting frequency and multiple root formation. Thomas [16] reported 86% rooting frequency for ‘Big Red’ in the presence of 2.9 µM 3-indoleacetic acid (IAA). Our study results are in agreement with those by Thomas [16], showing that rhubarb shoots rooted in vitro showed better shoot growth and higher survival rate after transfer to ex vitro conditions compared to direct rooting and acclimatization.

We found plantlets of ‘Malinowy’ showed a rapid increase in leaf size, petiole length and diameter when they were transferred to the greenhouse. Anthocyanin analyses revealed that ten-week old ‘Malinowy’ plantlets contained a very high amount of cyanidyn-3-O-rutinoside (176.7 mg·100 g^−1^) and cyanidin-3-O-glucoside (11.3 mg·100 g^−1^). Cyanidin derivatives were found previously to be the main anthocyanin in rhubarb stalk grown in the field. It has been demonstrated that rhubarb cultivars significantly differ in their anthocyanin content and percentage of the two main cyanidins [7,9]. The study presented by Takeoka et al. [9] showed that of twenty-one cultivars, total anthocyanin content ranged from 19.8 (‘Crimson Red’) to 341.1 mg·100 g^−1^ (‘Valentine’). Similarly, as in our study, cyanidyn-3-O-rutinoside was the main anthocyanin present in culinary rhubarb ‘Red Malinowy’ [11]. Moreover, in this genotype, twenty other phenolic compounds, including flavan-3-ols, flavonols and gallotannins, were identified. Among them, the most abundant was catechin (112 ng·mg^−1^ dry mass). The study of Clapa et al. [18] showed that phenolic composition can differ between in vitro and field-grown *Rheum rhabarbarum* plant extract. Further observations and analyses of phytochemical and nutritional compounds for rhubarb ‘Malinowy’ plants growing in vitro and in the field are needed.

## 4. Materials and Methods

### 4.1. Plant Material

Two donor plants of Polish garden rhubarb ‘Malinowy’ were carefully selected from a plantation in Kańczuga, located in the Subcarpathia Province, in southeastern Poland (WGS-84: 49°59′0″ N, 22°24′42″ E). Among other plants, they were distinguished by high yield and straight, thick and intensive red stalks. The selected plants were harvested at the beginning of November. Before culture initiation, the donor plants were tested by enzyme-linked immunosorbent assay (DAS-ELISA) with commercially available antibodies against ArMV, TuMV, *Cucumber mosaic virus* (CMV), *Cherry leaf roll virus* (CLRV), *Tobacco ring spot virus* (TRSV), *Tomato ring spot virus* (ToRSV), *Tomato spotted wilt virus* (TSWV), *Tomato black ring virus* (TBRV), *Strawberry latent ringspot virus* (SLRV) (LOEWE Biochemica, Sauerlach, Germany) and *Tobacco mosaic virus* (TMV) (Agdia, Elkhart, IN) by the method of Clark and Adams [44]. The plants were found to be virus free.

### 4.2. Culture Initiation

Shoot cultures were established from axillary buds isolated from dormant, eight-year-old rhizomes. First, they were divided into small parts then washed thoroughly with running tap water and soaked in fungicide. Buds were isolated and sterilized by soaking in commercial bleach (ace 4 mL/water 100 mL) for 20 min and then in 0.1% HgCl_2_ for 5 min. After rinsing in sterile water, the explants were placed in 50 mL Erlenmeyer flasks in Murashige and Skoog [45] (MS) medium containing different levels of nitrogen salts (100% and 75%), 100 mg·L^−1^ myo-inositol, vitamins (nicotinic acid, pyridoxine, thiamine (1.0 mg·L^−1^ each)), 2 mg·L^−1^ glycine, 88 mM sucrose and 6 g·L^−1^ agar (Biocorp, Poland). The effect of 4.4 µM benzylaminopurine (BAP) added together with 0.3 µM gibberellic acid (GA_3_) or 0.1 µM 1-naphthaleacetic acid (NAA) was studied. Full-strength MS medium without growth regulators was the control. The pH of the medium was adjusted to 5.8 before autoclaving. After 3 subcultures (each 3 weeks), the survival rate and the number of developed shoots were determined.

The shoots in all in vitro experiments were maintained at the temperature of 20 ± 2 °C under a standard 16/8 h photoperiod of 40 µmol m^−2^ s^−1^ (warm-white fluorescent lamps).

### 4.3. Shoot Multiplication

Murashige and Skoog medium with nitrogen salts reduced by a quarter containing 100 mg·L^−1^ myo-inositol, vitamins (nicotinic acid, pyridoxine and thiamine (1.0 mg·L^−1^ each) and 2 mg·L^−1^ glycine was used throughout the experiments. To obtain effective, cyclic shoot multiplication of rhubarb ‘Malinowy’, first, the effect of two adenine-type cytokinins (BAP and hydroxybenzylaminopurine (*meta*-topolin (mT)), added in concentrations of 4.4 µM, 6.6 µM and 12.4 µM, was examined (Experiment 1). After two subcultures (each lasting 4 weeks), the multiplication rate was determined. Then, the interaction between cytokinins (BAP or mT) and sucrose added at different concentrations (59, 73, 88, 117 and 175 mM) was studied. In Experiment 2, BAP was used at 6.6 µM and mT at 12.4 µM. After two subcultures, fresh mass, number and length of shoots, and number of leaves were determined. Leaf petioles were collected, lyophilized, and crushed into a homogenous powder using a laboratory mill (A 11, IKA, Staufen, Germany). The samples were then subjected to qualitative and quantitative analyses of sugars and anthocyanins by the HPLC method.

### 4.4. Rooting and Acclimatization

The aim of the experiments was to compare the efficacy of in vitro and ex vitro rooting and acclimatization. In the last subculture before rooting, shoots were grown in medium containing 4.1 µM *meta*-topolin.

For in vitro rooting, the selected shoots approximately 4 cm long were placed in modified MS medium containing 88 mM of sucrose and indole-3-butyric acid (IBA) at different concentrations (0, 0.49, 2.5 and 4.9 µM). After 3 weeks, rooting frequency, number of roots per explant and length of roots were determined. For the acclimatization experiment, shoots containing at least 5–6 roots were selected.

For ex vitro rooting and acclimatization, the shoots were taken directly from mT medium. The bases of shoots (approx. 4 cm long) were dipped in commercial rooting powder (Rhizopon AA 0.5%, Poland). Both types of microcutting (in vitro rooted and unrooted) were planted in multicellular trays 30 mm in diameter with a mixture of peat (Alonet Substrat, SIA Florabalt, LV-5106 Valle, Latvia) and perlite (2:1) in plastic plug boxes covered with transparent plastic caps to prevent dehydration. Ex vitro rooting and acclimatization took place in a growth room (25 ± 2 °C; PPFD—50 µmol m^−2^ s^−1^). The microcuttings were hardened by gradually decreasing air humidity. After 10 days, they were fed with 0.1% Kristalon (Yara Vlaardingen B.V., Netherlands) containing 18:18:18 (*w/w/w*) NPK. Four weeks after transfer to ex vitro conditions, the following data were collected: length of plantlets and the visual estimation of the root system on a three-point scale (1—no roots or single, short roots; 2—poorly developed root system, 3—well-developed root system, roots out of trays) (Figure 6).

After four weeks of acclimatization, in vitro rooted shoots were transferred to a greenhouse (at the end of February). They were grown at a temperature of 20/18 °C (day/night) with a 16 h photoperiod provided by additional lighting. Plants were manually watered and fed with 0.1% Kristalon. Plantlet growth (length of leaf petioles and leaf area) and biochemical status (soluble sugars and anthocyanin contents) were assessed after 1 and 6 weeks of growth in the greenhouse.

### 4.5. Soluble Sugar Analysis

The sugars were quantified by calibration curve for sucrose, glucose, fructose and sorbitol standards in the concentration range of 20–250 mg/100 mL, and the results are expressed as mg·100 g^−1^ dry mass (DM). An example chromatogram of sugar separation is shown in the Supplementary Materials (Figure S1). HPLC analysis of sugars was carried out with the HP 1200 system (Agilent Technologies, Waldbronn, Germany) equipped with an RI Detector with a BioRad Aminex-87C column (300 × 7.5 mm) according to European Standard EN 12630. The mobile phase was water purified by the MiliQ System (Milipore, Molsheim, France), isocratic flow was 0.6 mL·min^−1^ and column temperature was 80 °C. The lyophilized powder (100 mg) was extracted in 4 mL of redistilled water for 20 min in an ultrasonic bath. The suspension was then centrifuged (7000× *g*, 10 min). The resulting extract for sugar determination was filtered through a Sep-Pak^®^ PLUS C18 filter (Waters, Ireland).

### 4.6. Estimation of Anthocyanins by HPLC

HPLC analysis of anthocyanins was performed according to the method described by Nielsen et al. [46] with some modifications. In short, 5 µL of the eluate was analyzed using an Agilent HPLC Model HP 1200 equipped with a diode array detector (DAD). Separation was performed on a Phenomenex^®^Fusion RP column (250 mm × 4.6 mm; particle size = 4 µm) using a mobile phase consisting of water/ formic acid (95:5 *v/v*) (A) and acetonitrile (B). Elution profile: 0–16 min, 3%–9% B; 16–30 min, 12% B; 30–54 min, 33% B; 54–58 min, 90% B; 58–62 min, isocratic 90% B. The anthocyanins in the eluate were detected at 520 nm and a temperature of 25 °C. Their amounts were quantified by calibration with the standards of cyanidin-3-O-glucoside and cyanidin-3-O-rutinoside and expressed in mg·100 g^−1^ dry mass (DM). An example chromatogram of anthocyanins separation is shown in Supplementary Material (Figure S2).

The lyophilized powder (50 mg) was extracted in 2 mL of 60% methanol acidified with 1% formic acid for 20 min in an ultrasonic bath. The suspension was then centrifuged (7000× *g*, 10 min). The resulting extract for phenolic compound determination was filtered through a PTFE filter (0.45 µm, 15 mm).

### 4.7. Statistical Analysis

For the multiplication and in vitro rooting experiments, 30 shoots (6 shoots × 5 glass jars) were used in each treatment. For ex vitro rooting and acclimatization, 50 rooted and 50 unrooted shoots were used. The experiments were carried out twice. The final data were the means of the two replicated experiments. The data were subjected to one- (rooting and acclimatization experiment) or two-factor analysis of variance (ANOVA). The significance of the differences between means was evaluated by Duncan’s test at *p* = 0.05.

## 5. Conclusions

We developed a practical protocol for mass propagation of the selected genotype of rhubarb with a high content of anthocyanins. It enhanced the availability of phenolic-rich planting material for the establishment of commercial rhubarb plantations. The study indicates that growth and development of rhubarb shoots in vitro and secondary metabolite production are modulated by cytokinin and sucrose concentration. It was found that *meta*-topolin is a very useful cytokinin for rhubarb ‘Malinowy’ in vitro, which can be a good alternative for cultivars revealing multiplication and rooting difficulty in the presence of BAP. Although direct ex vitro rooting is possible, rhubarb shoots rooted in vitro showed better ex vitro establishment and growth in the greenhouse. Hence, in vitro rooting of rhubarb ‘Malinowy’ is preferred for mass production. Our results indicated that anthocyanins in leaf petioles of rhubarb plantlets were influenced by plant developmental stage and in vitro conditions. It is possible to elicit anthocyanins by high sucrose concentration combined with *meta*-topolin. Finally, the results obtained give important suggestions for introduction into the market and cultivation for rhubarb ‘Malinowy’, following the latest research conducted globally also on other interesting wild and cultivated plants [47,48,49].

## Figures and Tables

**Table 1:** Effect of mother (donor) plants on contamination of initial explants of Polish rhubarb ‘Malinowy’ in vitro.

Donor Plants	Total Number of Explants	Contaminated Explants (%)		Uncontaminated Explants that Did Not Developed Shoots (%)	Uncontaminated Explants that Developed Shoots (%)
		Bacteria	Fungi		
M1 *	34	29.4	-	3.0	67.6
M2	56	28.6	10.7	8.8	51.9

* ‘Malinowy’.

**Table 2:** Influence of nitrogen salts (75% and 100% MS medium) and growth regulators on the survival rate and initial shoot formation of rhubarb ‘Malinowy’ after 3 subcultures in the same fresh medium.

Propagation Medium	Survival Rate (%)	Shoot Number/Explant
100% MS control (hormone free)	0	-
100% MS + 4.4 µM BAP + 0.3 µM GA_3_	95.3	1.69 bc ^1^
100% MS + 4.4 µM BAP + 0.1 µM NAA	58.7	1.07 a
75% MS + 4.4 µM BAP + 0.3 µM GA_3_	94.1	1.92 c
75% MS + 4.4 µM BAP + 0.1 µM NAA	72.0	1.31 ab

^1^ Means marked with the same letter do not differ significantly (*p* = 0.05) according to Duncan’s test; the lowest value is marked with “a”.

**Table 3:** Effect of cytokinin type (BAP, mT) and sucrose levels on shoot formation, fresh mass of shoots, leaf number and shoot length of rhubarb ‘Malinowy’ after a 4-week subculture period.

Cytokinin Type	Sucrose Concentration (mM)	Fresh Mass (g)			Shoot Number	Shoot Length (mm)	Leaf Number/ Clump
		Shoot Clump	Callus	Single Shoot			
BAP	59	1.90 bc ^1^	0.170 a	0.395 a	4.8 e	31.6 a	25.1 d
	73	2.37 cd	0.162 a	0.566 b–c	4.3 de	37.5 bc	21.5 d
	88	1.68 ab	0.261 a–c	0.586 bc	3.1 bc	38.5 bc	16.9 c
	117	1.28 a	0.362 b–d	0.653 c–d	2.2 ab	42.4 cd	11.5 b
	175	1.41 a	0.532 e	0.907 e	1.5 a	49.0 e	8.9 ab
mT	59	2.51 d	0.256 a–c	0.446 ab	4.9 e	30.9 a	32.1 e
	73	2.31 d	0.237 ab	0.522 a–c	4.7 e	36.6 b	24.6 d
	88	1.61 ab	0.423 de	0.494 a–c	3.7 cd	40.1 b–d	12.7 b
	117	1.31 a	0.402 c–e	0.576 b–c	2.3 ab	38.9 bc	11.7 b
	175	1.28 a	0.430 de	0.768 de	1.7 a	44.8 de	6.9 a

^1^ Means indicated with the same letter within cytokinin type and sucrose levels do not differ significantly (*p* = 0.05) according to Duncan’s test; the lowest value is marked with “a”.

**Table 4:** Rooting response of rhubarb ‘Malinowy’ shoots after three-week growth in MS medium without auxin (control) and supplemented with IBA at different concentrations (0.49, 2.5, 4.9 µM).

Treatment	Auxin Concentrations (µM)	Rooting Frequency (%)	Root Number/Shoot	Root Length (mm)	Shoot Length (mm)
Control	0.0	40.0 a ^1^	2.1 a	29.2 b	75.2 a
IBA	0.49	55.9 b	2.5 a	28.8 b	72.0 a
	2.5	82.0 c	2.6 a	30.3 b	77.8 a
	4.9	94.9 d	10.7 b	13.84 a	78.5 a

^1^ Means of each parameter marked with the same letter do not differ significantly (*p* = 0.05) according to Duncan’s test; the lowest value is marked with “a”.

**Table 5:** Effect of microcutting status (rooted, unrooted) on survival, shoot growth and root system development of *Rheum* ‘Malinowy’ after 4 weeks of growth ex vitro (growing room).

Type of Microcutting	Survival Frequency (%)	Cutting Length (mm)	Rooting Index
Unrooted	83	72.8 a ^1^	1.9 a
Rooted	100	107.0 b	2.9 b

^1^ Means of each parameters indicated with the same letter do not differ significantly (*p* = 0.05) according to Duncan’s test; the lowest value is marked with “a”.

**Table 6:** Morphological and biochemical characteristics of *Rheum* ‘Malinowy’ during a 6-week growth period in the greenhouse.

Growth Duration	Length of Leaf Petioles (mm)	Leaf Area (cm^2^)	Soluble Sugar Contents (mg·100 g^−1^ DM)	Anthocyanin Contents (mg·100 g^−1^ DM)
1 week	60.5	20.2	Sucrose-t.a. Glucose-762 Fructose-858	cyanidin-3-O-glucoside-2.58 cyanidin-3-O-rutinoside-45.5
6 weeks	71.1	38.8	Sucrose-2196 Glucose-4958 Fructose-4790	cyanidin-3-O-glucoside-11.3 cyanidin-3-O-rutinoside-176.7

t.a.—trace amounts.

## Data Availability

The data presented in this study are available on request from the corresponding author.

## Supplementary Materials

The following are available online at https://www.mdpi.com/article/10.3390/plants10091768/s1, Figure S1: HPLC chromatograms of: (**A**) sugars in standard solution; (**B**) sugars in rhubarb ‘Malinowy’. Peak identification: 1—sucrose (Rt −8,1 min.), 2—glucose (Rt −9.8 min), 3—fructose (Rt −12.7 min), 4—sorbitol (Rt −21.4 min.). Figure S2: HPLC chromatograms of: (**A**) antocyanins in standard solution; (**B**) antocyanin in rhubarb ‘Malinowy’. Peak identification: 1—cyanidin-3-O-glucoside (Rt −22.3 min.), 2—cyanidin-3-O-rutinoside (Rt −25.7 min); Rt—retention time.
