# Supplementary material for: In Vitro Propagation Method for Production of Phenolic-Rich Planting Material of Culinary Rhubarb ‘Malinowy’
Source: Plants (Basel). 2021 Aug 25;10(9):1768. doi: 10.3390/plants10091768 (PMC8469749; doi:10.3390/plants10091768)
Supplement: Supplementary file 1 [file plants-10-01768-s001.zip › plants-1291209-supplementary.pdf]

plants

Supplementary materials

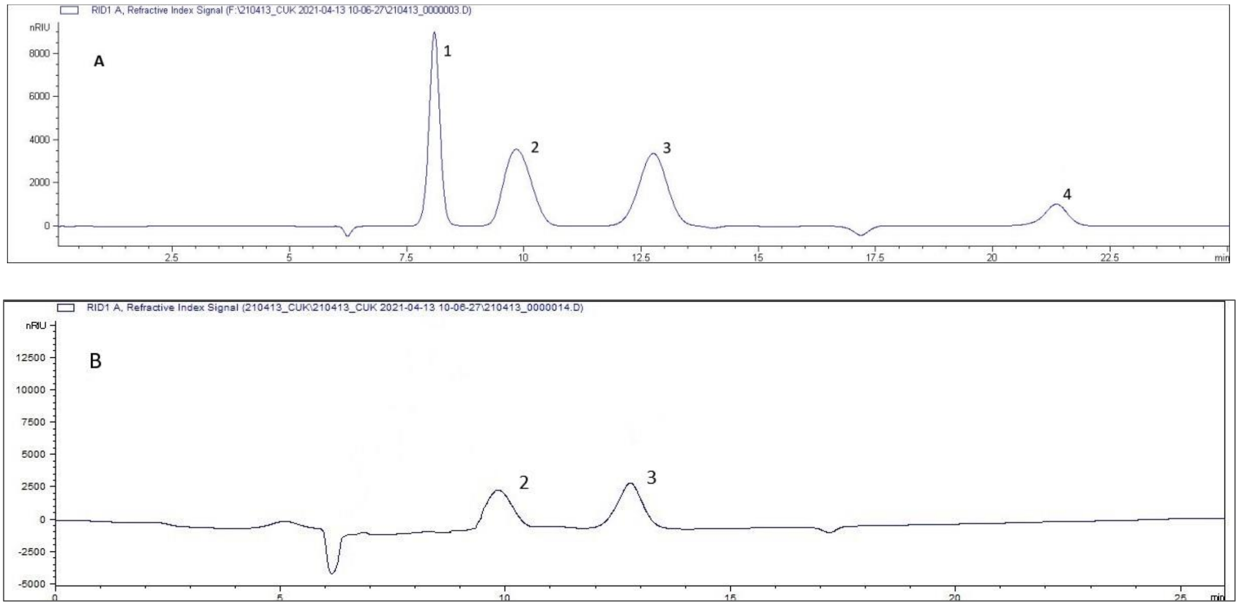

Figure S1. HPLC chromatograms of: (**A**) sugars in standard solution; (**B**) sugars in rhubarb 'Malinowy'. Peak identification: 1—sucrose (Rt −8,1 min.), 2—glucose (Rt −9,8 min), 3—fructose (Rt −12,7 min), 4—sorbitol (Rt −21,4 min.).

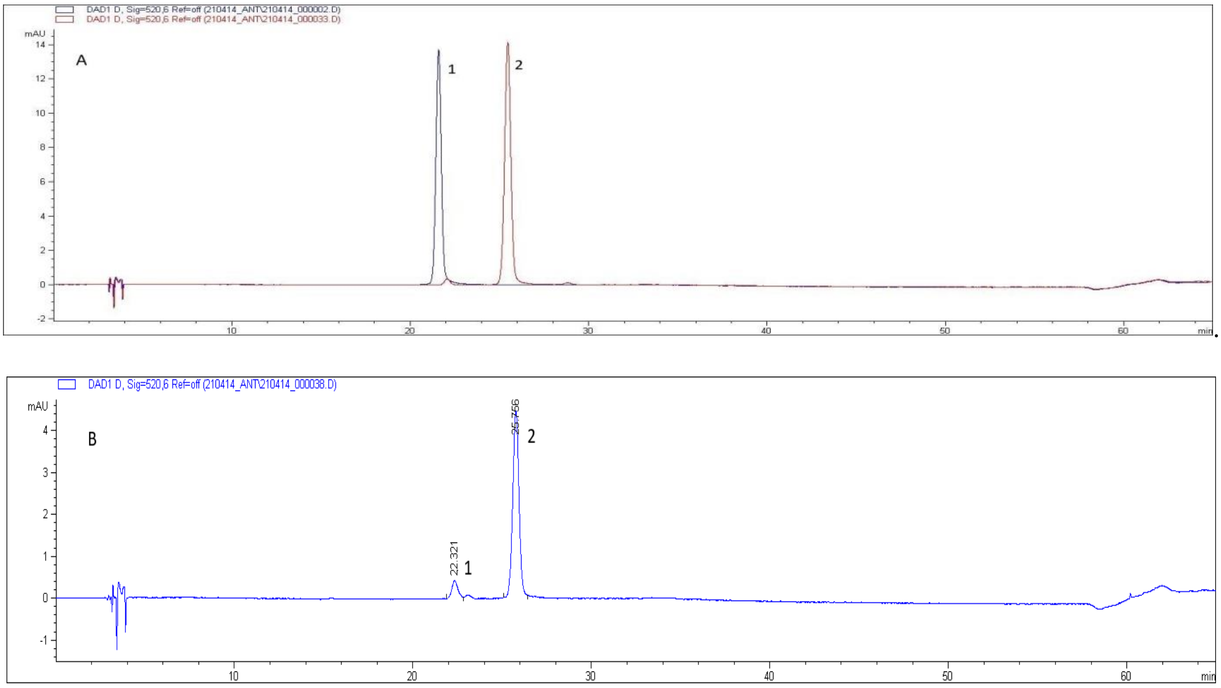

Figure S2. HPLC chromatograms of: (**A**) antocyanins in standard solution; (**B**) antocyanin in rhubarb 'Malinowy'. Peak identification: 1—cyanidin-3-O-glucoside (Rt −22,3 min.), 2—cyanidin-3-O-rutinoside (Rt −25,7 min); Rt—retention time.
